# Social determinants and work-related musculoskeletal disorders in Brazil

**DOI:** 10.1371/journal.pone.0306840

**Published:** 2024-07-15

**Authors:** Nayara da Silva Pontes, Sanderson José Costa de Assis, Gabrielle Silva de Oliveira, Rebeca de Castro Santana, Rebeca Freitas de Oliveira Nunes, Emannuel Alcides Bezerra Rocha, Clécio G. de Souza, Angelo Giuseppe Roncalli, Marcello Barbosa Otoni Gonçalves Guedes

**Affiliations:** 1 Federal University of Rio Grande do Norte, Rio Grande do Norte, Brazil; 2 Public Health Program, Federal University of Rio Grande do Norte, Rio Grande do Norte, Brazil; 3 Uninassau University Center, Rio Grande do Norte, Brazil; 4 Faculty of Health Sciences of Trairi, Santa Cruz, Federal University of Rio Grande do Norte, Rio Grande do Norte, Brazil; 5 Graduate Program in Physical Therapy, Federal University of Rio Grande do Norte Natal, Rio Grande do Norte, Brazil; UNESP: Universidade Estadual Paulista Julio de Mesquita Filho, BRAZIL

## Abstract

This study aimed to analyze the prevalence of work-related musculoskeletal disorders (WMSD) and their association with individual and contextual factors in the Brazilian population. This quantitative cross-sectional study used secondary data from the Brazilian National Health Survey from August 2013 to February 2014. The dependent variable included WMSD, and independent variables were analyzed as individual and contextual factors. WMSD was mostly prevalent in females, individuals aged 43 to 59 years, with chronic physical or mental disorders, reporting frequent sleep disorders, and performing integrative and complementary health practices, physical exercise or sports, and heavy physical activity or housework. Regarding contextual factors, high social classes and proportion of individuals with formal work were associated with a high prevalence of WMSD, whereas a high Gini index was associated with a low prevalence. Thus, a high prevalence of WMSD in the Brazilian population was associated with individual and contextual factors, which should be the target of health professionals for actions of promotion, prevention, and intervention at individual or collective care levels.

## Introduction

Work-related musculoskeletal disorders (WMSD) are conditions with several symptoms and etiologies resulting from repetitive motions and excessive use of muscle groups [1, 2]. They are typically associated with inadequacies at work (e.g., long working hours and non-ergonomic postures), manifested by previous health conditions (e.g., joint and circulatory problems), or personal factors (e.g., motivational aspects, financial encouragements, and interpersonal relationships at work) [3–7].

WMSD are highly prevalent worldwide and represent a significant health problem [6]. According to the Global Burden of Disease, musculoskeletal disorders present a high prevalence and burden [8–10], affecting about 1.71 billion people and contributing to disability and human suffering [11]. Also, musculoskeletal disorders have become the third leading cause of years lived with disability over the past 30 years [12]. Poor occupational conditions related to the environment and tasks [1, 13] and biological and physiological determinants (e.g., advanced age, anxiety, and stress) may be risk factors for WMSD [14]. In addition, other contextual factors related to access and quality of healthcare services may be involved in the diagnosis of WMSD, highlighting the importance of a biopsychosocial assessment and management [15]. Thus, access to healthcare services should be expanded to increase diagnoses and treatment of WMSD [16].

WMSD affect adults of both sexes during the most productive phase of life, which may reduce productivity and attendance at work and increase financial costs related to compensations and functional disabilities for private and public institutions, including retirement due to disability [6, 13]. In this sense, population-based studies should address the influence of contextual and individual factors, especially in developing countries with significant socioeconomic and cultural diversity, such as Brazil. These studies might support health systems worldwide in planning public policies for prevention and intervention actions in the workplace [17]. Therefore, this study aimed to analyze the prevalence of WMSD and its association with individual and contextual factors in the Brazilian population.

## Materials and methods

This quantitative cross-sectional study used secondary data from the Brazilian National Health Survey (PNS) from August 2013 to February 2014, organized by the Brazilian section of the United Nations Development Programme (PUND) and National Registry of Health Establishments (CNES).

The PNS is a population-based survey with three questionnaires conducted by the Brazilian Ministry of Health with the Brazilian Institute of Geography and Statistics (IBGE) to characterize the health status and lifestyle of the national population, including use and access to healthcare services. The first questionnaire collected information on household characteristics following the demographic census and National Household Sample Survey (PNAD) standards. The second questionnaire addressed all household members, extending the Health Supplement of the PNAD. The individual questionnaire focused on major non-communicable chronic diseases (NCD), lifestyle, and access to healthcare, and was responded by one household resident aged ≥ 18 years (selected using equiprobability among all eligible residents) [18, 19].

PNUD is an organization aiming to fight against poverty and promote development by providing technical support to partners using various methodologies and expertise, particularly in socioeconomic information of countries. In addition, the CNES provides data on structural capacity of buildings, available services, professionals working in health units, financial information, and other data from all states and municipalities in the country.

The target population of the PNS were residents of private households in the national territory. The sampling comprised a three-stage cluster design with stratification of primary sampling units: census tracts comprised the primary-stage units, households comprised the second-stage, and residents aged ≥ 18 years comprised the third-stage. Of 64,348 households in the final sample of the 2013 PNS, 60,202 individual interviews were conducted and selected in this study [20]. However, individuals aged ≥ 60 years were excluded to analyze only the economically active age group, resulting in a sample size of 49,024 individuals.

The 2013 PNS was approved by the National Commission of Ethics in Research of the National Health Council (no. 328,159) on June 26, 2013. All individuals were informed about the survey aims and signed an informed consent form, and the survey followed guidelines for human studies and the Declaration of Helsinki [19].

WMSD were the dependent variable of this study and characterized by a positive response (options of “yes” or “no”) to the following question in the PNS: "has any physician ever diagnosed you with a WMSD?". Independent variables were divided into individual (i.e., biological, lifestyle, social networks, and community) and contextual factors (i.e., socioeconomic, cultural, and environmental) extracted from the 2013 PNS (Table 1).

**Table 1:** Dependent and independent (individual and contextual factors) variables of the study.

	Variable	Description	Original category (new category)
Dependent	Work-related musculoskeletal disorders	Individuals were asked if a physician had already diagnosed the work-related musculoskeletal disorder	Yes or no (no modification)
Independent (Individual factors)	Sex	Sex of the individuals	Male or female (no modification)
	Age	Age (in years)	Numerical variable, categorized by tertiles: ≤ 31 years; from 32 to 42 years; and ≥ 43 years
	Marital status	Marital status was asked during the interview	Categorical variable, categorized as single or not single
	Can read and write	Individuals were asked if they could read and write	Yes or no (no modification)
	Health, medical, or dental insurance	Individuals were asked if they had health, medical, or dental insurance	Yes or no (no modification)
	Prevented from conducting any usual activity due to health issues	Prevented from conducting any usual activity due to health conditions	Yes or no (no modification)
	Diagnosis of any chronic physical or mental disorder	Individuals were asked about having a diagnosis of any chronic or long-term physical or mental disorder	Yes or no (no modification)
	Use of integrative and complementary practices in health	Individuals were asked if they performed any integrative and complementary practice	Yes or no (no modification)
	Days with sleep problems	Individuals were asked about the frequency (days) of sleep problems	The frequency of sleep problems was categorized as no day, less than half the days, more than half the days, and almost every day
	Days with fatigue-related problems	Individuals were asked about the frequency (days) of fatigue-related problems during the day	The frequency of fatigue-related problems was categorized as no day, less than half the days, more than half the days, and almost every day
	Depressed days	Individuals were asked about the frequency (days) that they felt depressed	The frequency that they felt depressed was categorized into no days and some days
	Practice of physical exercise or sports	Individuals were asked whether they practiced any physical exercise or sports	Yes or no (no modification)
	Heavy physical activity or housework	Individuals were asked whether they perform heavy housework or other physically demanding activities	Yes or no (no modification)
	Diagnosis of depression	Individuals were asked if a physician or mental health professional diagnosed them with depression	Yes or no (no modification)
Independent (contextual factors)	Gini Index	Evaluate the income (from 0 to 1); low scores represent low inequality	Numerical variable, categorized by tertiles: ≤ 0.56; from 0.57 to 0.61; and ≥ 0.62
	Proportion of individuals with formal work	Measurement of formally employed individuals (i.e., employed with a signed work contract)	Numeric variable, categorized by tertiles: ≤ 45.53; from 45.54 to 62.00; and ≥ 62.01
	Social class	Classification by the Brazilian Institute of Geography and Statistics with five income classes (A, B, C, D, and E); those closer to A present higher income	Numeric variable, categorized by tertiles: E and D classes; C class; and A and B classes
	Family Health Strategy coverage	A primary care action to expand, enhance, and consolidate healthcare to guide the work process, promoting expansion, effectiveness, and impact on individual and collective health. This action requires the presence of a family health team composed of multidisciplinary professionals. The evaluation of this variable was carried out at the state level.	Numeric variable, categorized by tertiles
	Family Health Support Nucleus coverage	This support nucleus aims to broaden the scope of primary healthcare actions, ensuring quality and service resolution. The teams are composed of integrated multidisciplinary professionals to support the family health teams and those attending specific populations, such as riverside and riverine communities. The evaluation of this variable was carried out at the state level.	Numeric variables categorized by tertiles

Individual factors comprised sex, age, sleep and fatigue-related problems, practice of physical exercise or sports, and heavy physical activity or housework. Also, individuals were categorized into three age groups based on the tertiles of the studied sample (≤ 31 years, 32 to 42 years, and ≥ 43 years).

Contextual factors included the Gini index (i.e., measurement of social inequality through income concentration within a particular group), proportion of individuals with formal work (i.e., number of formally employed individuals with signed work contracts), and social class (i.e., classification of a society based on economic, political, and cultural characteristics). The Gini index was calculated based on the state of residence of each individual. The IBGE categorizes social classes into five income brackets (A, B, C, D, and E), in which the income increases from E to A. These variables presented plausible theoretical associations with the WMSD and were obtained from the PUND database and categorized according to states by the national census of the IBGE. Also, the following variables related to health services were extracted from the CNES database: proportion of Family Health Strategy (ESF) and Family Heath Support Nucleus (NASF). All quantitative variables were classified into three categories based on their respective tertiles (Table 1).

**Fig 1:**
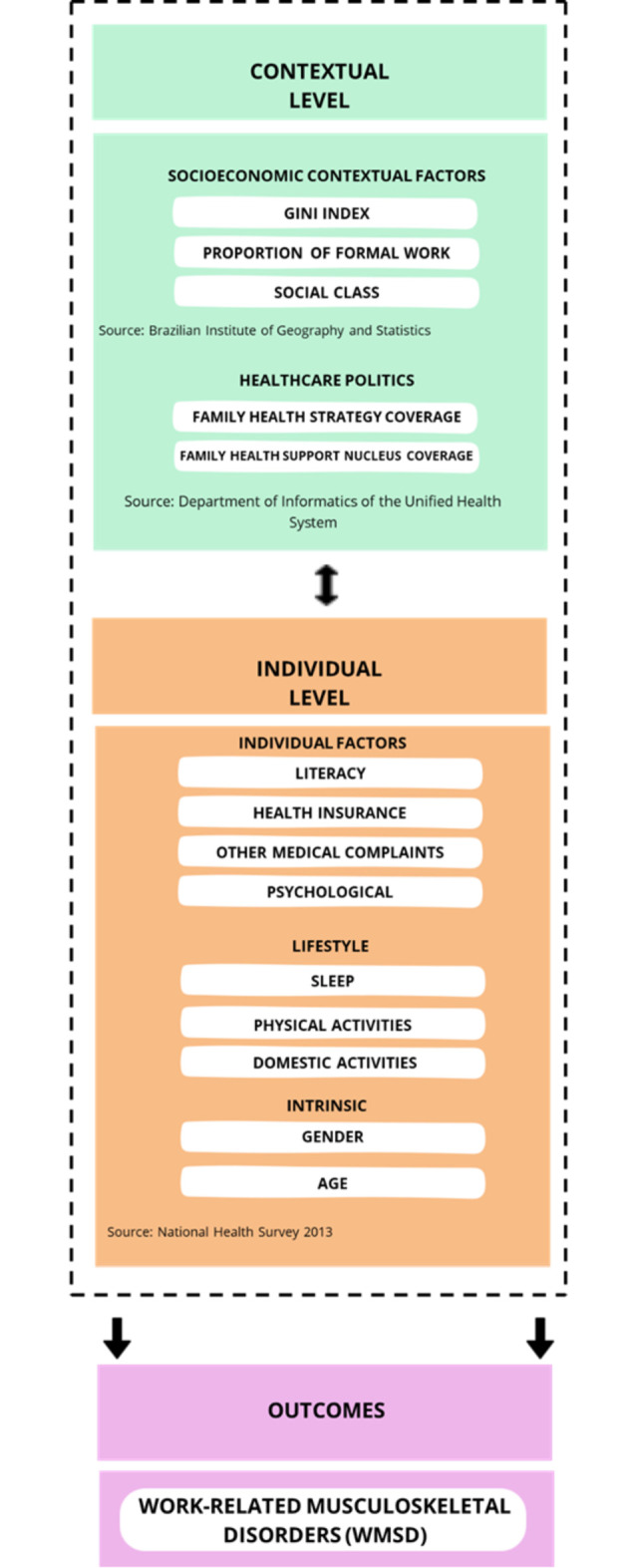
Study framework, Brazil, 2013.

The theoretical modeling was based on the social determinants of health model proposed by Dahlgren and Whitehead [21], in which different layers represent specific levels of determination. Thus, the closest level refers to individual characteristics, while distant level consists of socioeconomic, cultural, and environmental determinants (e.g., contextual factors for each individual) (Fig 1) [21, 22].

Multilevel modeling was used to assess the influence of individual and contextual factors on WMSD, in which contextual factors may be considered as social aggregates due to their effect on community. Thus, individuals may be considered as the first level (lowest), and their residences as the second level (outer).

Descriptive analysis was performed to determine cutoff points or criteria for categorization. Three categories based on tertiles were determined for metric variables to distribute the number of participants per category better. Association tests (e.g., Rao-Scott chi-square test) were conducted between the WMSD and all independent variables, and those with p ≤ 0.2 were included in the multiple regression analysis. Also, an unadjusted prevalence ratio (PR) and 95% confidence interval (95% CI) were initially estimated.

A multilevel Poisson regression model incorporated variables from different levels. This model was used because the odds ratio estimated through logistic regression may present overestimated values when working with common outcomes. Initially, the modeling started with a null model to assess the viability of the multilevel approach, and estimate the basic partition between levels before including individual and contextual variables. Subsequently, variables from all dimensions were included, starting with the proximal ones, until the inclusion of the most distal ones. Model 1 was adjusted for variables at the individual level, while in model 2 contextual variables were added. The final "complete" model was the model that included individual, contextual variables and variables related to health services. The model adjustment was carried out using the Likelihood Ratio Test, as well as by observing the change in variances between each model. Finally, an interaction term between contextual and individual factors was created to analyze the presence of cross-level interaction. Significance level was set at 5% (α < 0.05).

## Results

Of 64.348 households included in the 2013 PNS, 60.202 Brazilian individuals (≥ 18 years old) of both sexes residing in urban and rural areas across all states were interviewed. However, individuals aged ≥ 60 years were excluded from the final sample of the present study, resulting in 49.024 individuals.

Table 2 shows the descriptive analysis and initial associations based on bivariate analysis. A high prevalence of WMSD was identified in women, individuals aged between 43 and 59 years, and performing heavy physical activity or housework.

**Table 2:** Relationship between the dependent variable work-related musculoskeletal disorders and independent variables of the study.

	Work-related musculoskeletal disorders			
	No	Yes	p-value	PR (95% CI)
	n (%)	n (%)		
**Sex**				
Male	21,075 (98.6)	289 (1.4)		1
Female	26,945 (97.4)	715 (2.6)	< 0.0001	1.91 (1.67–2.19)
**Age**				
≤ 31 years	17,115 (98.9)	193 (1.1)		1
32 to 42 years	14,617 (97.9)	321 (2.1)	< 0.0001	1.86 (1.56–2.23)
≥ 43 years	16,288 (97.1)	490 (2.9)	< 0.0001	2.42 (2.05–2.86)
**Prevented from conducting any usual activity due to health issues**				
No	44,330 (98.2)	793 (1.8)		1
Yes	3,690 (94.6)	211 (5.4)	< 0.0001	3.08 (2.65–3.59)
**Days with sleep problems**				
No day	34,500 (98.6)	506 (1.4)		1
Some days	13,520 (96.4)	498 (3.6)	< 0.0001	2.45 (2.17–2.78)
**Practice of physical exercise or sports**				
No	32,869 (98.2)	616 (1.8)		1
Yes	15,151 (97.5)	388 (2.5)	< 0.0001	1.27 (1.11–1.44)
**Heavy physical activity or housework**				
No	39,185 (98.3)	686 (1.7)		1
Yes	8,835 (96.5)	318 (3.5)	< 0.0001	1.95 (1.71–2.23)
**Diagnosis of depression**				
No	44,957 (98.3)	785 (1.7)		1
Yes	3,063 (93.3)	219 (6.7)	< 0.0001	0.30 (0.26–0.35)
**Gini index**				
≤ 0.56	19,975 (97.1)	607 (2.9)		1
0.57 to 0.61	19,943 (98.5)	299 (1.5)	-	-
≥ 0.62	8,102 (98.8)	98 (1.2)	-	-
**Proportion of individuals with formal work**				
≤ 45.53	15,586 (98.9)	176 (1.1)		1
45.54 to 62.00	15,830 (98.4)	259 (1.6)	-	-
≥ 62.01	16,604 (96.7)	569 (3.3)	-	-
**Social class**				
E and D	18,154 (98.4)	286 (1.6)		1
C	19,150 (98.0)	392 (2.0)	-	-
A and B	10,716 (97.0)	326 (3.0)	-	-

PR: prevalence ratio; 95% CI: 95% confidence interval.

In the multilevel analysis, a high prevalence of WMSD was observed in females (PR = 1.52; 95% CI = 1.31 to 1.75), individuals aged between 43 and 59 years (PR = 2.12; 95% CI = 1.80 to 2.52), with frequent sleep problems (PR = 1.98; 95% CI = 1.66 to 2.36), and performing physical exercise or sports (PR = 1.48; 95% CI = 1.30 to 1.69) and heavy physical activity or housework (PR = 1.57; 95% CI = 1.37 to 1.80) (Table 3).

**Table 3:** Multilevel Poisson regression analysis for work-related musculoskeletal disorders according to individual and contextual factors.

	Null model	Model 1 (n = 49.024)		Model 2 (n = 49.024)		Final model (n = 49.024)	
Factors	(n = 49.024)	PR (95% CI)	p-value	PR (95% CI)	p-value	PR (95% CI)	p-value
**First Level (individual)**							
Sex							
Male		1		1		1	
Female		1.53 (1.32–1.76)	< 0.001	1.51 (1.31–1.75)	< 0.001	1.52 (1.31–1.75)	< 0.001
Age							
≤ 31 years		1		1		1	
32 to 42 years		1.73 (1.45–2.07)	< 0.001	1.73 (1.44–2.07)	< 0.001	1.73 (1.44–2.07)	< 0.001
≥ 43 years		2.11 (1.78–2.50)	< 0.001	2.12 (1.79–2.52)	< 0.001	2.12 (1.80–2.52)	< 0.001
Prevented from conducting any usual activity due to health issues							
No		1		1		1	
Yes		2.01 (1.71–2.36)	< 0.001	2.02 (1.73–2.38)	< 0.001	2.02 (1.72–2.38)	< 0.001
Days with sleep problems							
No day		1		1		1	
Less than half the days		1.56 (1.33–1.85)	< 0.001	1.57 (1.33–1.85)	< 0.001	1.57 (1.33–1.85)	< 0.001
More than half the days		1.74 (1.39–2.18)	< 0.001	1,76 (1.40–2.21)	< 0.001	1.76 (1.40–2.21)	< 0.001
Almost every day		1.97 (1.65–2.35)	< 0.001	1.98 (1.66–2.36)	< 0.001	1.98 (1.66–2.36)	< 0.001
Practice of physical exercise or sports in the last three months							
No		1		1		1	
Yes		1.52 (1.33–1.73)	< 0.001	1.48 (1.30–1.58)	< 0.001	1.48 (1.30–1.69)	< 0.001
Heavy physical activity or housework							
No		1		1		1	
Yes		1.56 (1.36–1.79)	< 0.001	1.57 (1.37–1.80)	< 0.001	1.57 (1.37–1.80)	< 0.001
Diagnosis of depression							
No		1		1		1	
Yes		0.53 (0.45–0.63)	< 0.001	0.54 (0.45–0.63)	< 0.001	0.54 (0.46–0.63)	< 0.001
**Second Level (contextual)**							
Gini index							
≤ 0.56				1		1	
0.57 to 0.61				0.70 (0.49–1.00)	0.050	0.75 (0.54–1.05)	0.093
≥ 0.62				0.48 (0.31–0.75)	0.001	0.56 (0.38–0.83)	0.004
Proportion of individuals with formal work							
≤ 45.53				1		1	
45.54 to 62.00				0,87 (0.60–1.27)	0.486	1.16 (0.99–1.35)	0.065
≥ 62.01				1.80 (1.16–2.80)	0.008	1.40 (1.19–1.65)	0.001
Social class							
E and D				1		1	
C				1.15 (0.99–1.34)	0.071	1.16 (0.99–1.35)	0.065
A and B				1.40 (1.18–1.65)	< 0.001	1.40 (1.19–1.65)	< 0.001
Proportion of Family Health Strategy (per 100,000 inhabitants)							
≤ 6,447						1	
5,552 to 6,446						1.17 (0.87–1.59)	0.302
≥ 5,552						1.39 (1.05–1.85)	0.021
Proportion of Family Health Support Nucleus (per 100,000 inhabitants)							
≥ 1.877						1	
0.946 to 1.876						0.93 (0.67–1.28)	0.655
≤ 0.946						0.69 (0.49–0.96)	0.028
** Random effects**							
Variance (95% CI)		0.26 (0.13–0.53)		0.58 (0.017–0.19)		0.045 (0.013–0.16)	
Changes in variation (%)				123.08		92.24	
Likelihood ratio test (Chi-square test, p-value)		121.96 (< 0.001)		6.87 (0.044)		5.64 (0.0088)	

PR: prevalence ratio; 95% CI: 95% confidence interval. Model 1: adjusted for individual-level variables. Model 2: adjusted for variables at individual and contextual levels. Final model: adjusted for individual, contextual and health service-related variables.

Regarding contextual factors, the high Gini index was associated with low prevalence of WMSD (PR = 0.56; 95% CI = 0.38 to 0.83). Also, social classes A and B presented higher prevalence of WMSD (PR = 1.40; 95% CI = 1.18 to 1.65) than E and D, whereas class C presented no association (p = 0.071). A high proportion of individuals with formal work was also associated with high prevalence of WMSD (PR = 1.40; 95% CI = 1.19 to 1.65) (Table 3). In addition, a low proportion of ESF was associated with high prevalence of WMSD (PR = 1.39; 95% CI = 1.05 to 1.85), whereas a high proportion of NASF was associated with a low prevalence of WMSD (PR = 0.69; 95% CI = 0.49 to 0.96).

## Discussion

This study aimed to analyze the prevalence of WMSD and its association with individual and contextual factors in the Brazilian population using data from the 2013 PNS, being a pioneer national study on this topic. Female sex, age, frequent sleep problems, and practice of physical exercise or sports and heavy physical activity or housework were some individual factors associated with a high prevalence of WMSD. Regarding contextual factors, high social classes and proportion of individuals with formal work were associated with a high prevalence of WMSD, whereas a high Gini index was associated with a low prevalence. Also, municipalities with low proportion of ESF and high NASF presented high prevalence of WMSD.

The high prevalence of WMSD in females corroborated other studies [1, 23] and might be due to the double or triple workloads they perform nowadays, including commitment to housework, children, and work outside home. Also, the combination of domestic and occupational activities might increase exposure to risk factors for WMSD (e.g., repetitive motions, non-ergonomic postures, prolonged standing, and lack of rest time) [24, 25]. Another explanation could be the anatomo-physiological differences between female and male bodies (e.g., height, body mass, musculoskeletal composition, and vulnerability of joints), possibly resulting in physical overload and WMSD [23, 26, 27]. Moreover, women search and use more healthcare services than men, resulting in more diagnoses of WMSD [28, 29].

The prevalence of WMSD increased with age in this study. Considering that individuals ≥ 60 years old (i.e., regular age for work retirement) were excluded, this result was related to the oldest age group assessed (i.e., 43 to 59 years), which was composed of individuals economically active and productive, corroborating other studies [15, 30]. Older adults may be more vulnerable to occupational and ergonomic risk factors (e.g., long exposure time, inappropriate workstations, exposure to vibrations and mechanical pressures, task variability, cognitive demands, and organizational and psychosocial pressures) than younger adults [30]. Therefore, aging under adverse working conditions may lead to social exclusion, illness, and pain [14, 15, 31].

Individuals in high social classes and with private health insurance presented high prevalence of WMSD, which might be explained by their easy access to healthcare services, increasing the diagnosis of WMSD [32–34]. The Gini index was a contextual factor measuring the income concentration in a group with scores ranging from 0 to 1; low scores represent income equality, whereas high scores represent inequality. In this sense, high scores on this index were associated with a low prevalence of WMSD in this study. Although individuals in areas with lower income equality usually need more healthcare than those in areas with higher equality, they have lower access to these services [33, 35]. Thus, considering that WMSD is diagnosed through specialized care [16], individuals residing in areas with income inequality might present lower prevalence of WMSD than those in areas with income equality due to underdiagnosis related to access to healthcare services [35].

In contextual factors, high proportion of individuals with formal work was associated with a high prevalence of WMSD, which might be explained by the modern work processes to which professionals with formal contracts are exposed. Also, a high proportion of individuals with informal work might result in socioeconomically vulnerable groups with low access to healthcare services, resulting in low frequency of WMSD diagnosis, whereas those with formal work may have easy access to these services and diagnosis [36]. Another hypothesis is that formal work environments with occupational health professionals might facilitate WMSD diagnosis since workers need the diagnosis to receive labor compensation, sick leave, or disability retirement. However, this hypothesis still needs to be studied. Few studies have investigated the relationship between work formalization and prevalence of WMSD and the influence of occupational health professionals in diagnosing the condition. Thus, future research should address this topic by investigating the prevalence of WMSD in formal and informal work environments and the relationship between its diagnosis and the presence of occupational health professionals in the workplace.

Primary healthcare in Brazil comprises individual and collective health actions (e.g., health promotion and protection, disease prevention, early diagnosis, treatment, rehabilitation, harm reduction, and health maintenance), in which the ESF and the NASF are important programs for this care [37]. The ESF provides and expands access to public healthcare services in Brazil, being responsible for managing mild- to moderate-severity health conditions, including WMSD [38]. The present results indicated that reduced coverage of this program may lead to a high prevalence of WMSD. The lack of preventive actions and care from the ESF may be the main factor in this relationship. Also, Brazilian population-based research indicated a positive impact of ESF on the diagnosis of other outcomes [39, 40]. Thus, studies with other methodological designs should assess the relationship between WMSD and ESF coverage.

The main focus of NASF is the qualification and support of primary healthcare services, ensuring specialized management and increased effectiveness [37]. The present study indicated a relationship between high proportion of NASF and prevalence of WMSD. The increased NASF coverage might have enhanced the qualification of primary healthcare teams and diagnosis of WMSD in these areas [41]. Also, important tools (e.g., protocols for user regulation with reference and counter-reference to diagnostic services in the healthcare network) are frequently used in areas with NASF coverage, providing an spatiotemporal analysis of health indicators [42]. In this sense, studies directly analyzing these factors may confirm the hypothesis.

WMSDs have historically received limited attention from researchers, largely due to their low fatality rates and perceived irreversibility [43]. Furthermore, global health systems often lack sufficient resources to effectively address the prevention and management of non-communicable diseases (NCDs) [44]. Despite the significant global burden attributed to WMSDs and the well-established correlation between pain and disability, these conditions have not been adequately integrated into the non-communicable disease prevention and management policies of the World Health Organization (WHO) across high-, middle-, and low-income countries [14, 15]. Also, most countries (63% to 83%) had policies focusing on cancer, cardiovascular diseases, diabetes, and respiratory diseases, and musculoskeletal conditions had low attention [17]. Consequently, the lack of emphasis on early diagnosis and treatment, which are pivotal strategies for preventing disabilities and improving the quality of life of individuals with WMSDs [45].

Individuals who reported frequent sleep problems showed a high prevalence of WMSD. This result corroborated a previous study [46] showing that poor self-perception of sleep quality was associated with a high prevalence of musculoskeletal pain. However, it should be interpreted with caution since the study design [46] did not directly establish a cause-and-effect relationship. Other studies suggested that pain from WMSD may lead to sleep-related disorders [47, 48]. Also, considering that sleep provides functional recovery of musculoskeletal structures subjected to high loads and tensions during the day, sleep impairment may cause a pathological cycle and explain the high prevalence of WMSD in people with sleep problems reporting fatigue throughout the day [46].

Performing physical exercise or sports was associated with high prevalence of WMSD. However, this finding diverged from other studies [49, 50] showing that regular physical and leisure activities and workplace gymnastics may reduce the risk of musculoskeletal complaints. Physical exercise was also associated with reduced impairments in professional performance, exposure to musculoskeletal symptoms, and greater functional capacity in activities of daily living and work [51, 52]. However, the exercise type and methods were not assessed in these studies, and some factors (e.g., intensity and frequency of exercises) may directly impact the health of individuals with WMSD. In addition, individuals with WMSD might have sought or been advised to perform physical activity to achieve its benefits, which could explain the high prevalence of these conditions among physically active populations and justify the divergent findings observed in the present study. However, further studies with different methodological designs and analyses are needed to clarify this hypothesis.

This study had some limitations. The WMSD may have been underestimated since its identification depends on clinical diagnosis, which is subjected to the level, quality, and type of access to healthcare services. Memory bias and inadequate resources to assess data quality were also limitations due to the nature of epidemiological surveys. However, these limitations do not invalidate the findings of this study but stimulate further research to complement and deepen the discussion on the topic. Considering the cross-sectional design of this study, the reverse causality could not be explored, and some variables may lose context. However, these variables may separately explain some outcomes and clarify which factors should be investigated in WMSD. Also, the use of multilevel multivariate modeling reduced confusion biases between variables. Although the results met the proposed aims, some potential confounding variables could not be assessed, such as the quality of work, biomechanical variables (e.g., repetitive motion at work), and non-ergonomic postures in the work environment of individuals with WMSD. Thus, these factors should be considered in future studies.

Although the analyzed variables had a wide diversity, they are present in many professions of individuals with WMSD, especially in activities performed jointly with formal works. For example, women may be more exposed to these variables than men due to triple workload, and equity at work may be a determining factor in reducing WMSD. Men and women are exposed to physical and mental factors at work, however women are more commonly involved in jobs that involve other factors, such as irregular work shifts, time pressure and interpersonal relationships, which can increase the prevalence of WMSDs in this population [53]. The present findings may be powerful tools to develop public policies for WMSD and its associated factors. Considering that Brazil has continental dimensions with a healthcare system mainly based on the provision of public services, developing countries or those with similar healthcare models may also benefit from the results and hypotheses investigated in this study.

## Conclusion

It was observed in this study that contextual factors such as greater formal employment, access to health services, higher social class and lower Gini index were associated with a higher prevalence of WMSDs, in addition to factors such as female sex and working age. However, variables directly related to work, such as the quality of work of people with WMSDs, biomechanical variables such as excessive repetition at work and inadequate postures in the work environment, were not investigated. These factors must be evaluated together in new studies, as they can be the target of attention from professionals at individual or collective levels of care for promotion, prevention and intervention actions.

## Supporting information

S1 File (PDF)
