# Supplementary material for: Social determinants and work-related musculoskeletal disorders in Brazil
Source: PLoS One. 2024 Jul 15;19(7):e0306840. doi: 10.1371/journal.pone.0306840 (PMC11249243; doi:10.1371/journal.pone.0306840)
Supplement: S1 File — (PDF) [file pone.0306840.s001.pdf]

Request for Authorship Change(s)

Authorship change requests are subject to PLOS approval. All applicable sections of this form must be completed in full before we will review your request. Please note that per the PLOS Authorship Policy we require agreement from all authors (including those being added or removed) for any changes to a submission's author list.

PLOS does not generally consider requests to add or remove authors after editorial acceptance; any late-stage requests require approval by the journal's editorial team.

In some cases, depending on the nature, timing, and/or extent of authorship changes requested, PLOS may require verification of the updated author list and contributions by an official at the authors' institution(s). Difficulties reaching an institutional official may delay your submission's peer review or publication.

Section 1. Confirm adherence to PLOS policies

☐ ☒ Check here to confirm you have read the *PLOS* Authorship policy, and that the updated author list requested in this form complies in full with the criteria outlined in the Authorship Requirements section of our policy. Key points from the policy that pertain to authorship criteria are summarized here:

Everyone listed as an author must meet all criteria for authorship, and everyone who meets the criteria for authorship should be listed as an author.

Authorship criteria for *PLOS Medicine*:

1. Conception and design of the work, acquisition of data, or analysis and interpretation of data; and
2. Drafting the article or revising it critically for important intellectual content; and
3. Final approval of the version to be published; and
4. Agreement to be accountable for all aspects of the work

Authorship criteria for all other PLOS journals:

1. Substantial contributions to one or more of the following: conception or design of the work; acquisition, analysis, or interpretation of data; creation of new software used in the work; drafting or substantially revising the article; and
2. Approved the submitted version (and any substantially modified version that involves the author's contribution to the study); and
3. Agrees to be personally accountable for the author's own contributions AND to ensure that questions related to the accuracy or integrity of any part of the work are appropriately investigated, resolved, and the resolution documented in the literature.

Any contributions that do not meet authorship criteria should be discussed in the Acknowledgments section of the manuscript; authors are responsible for obtaining permission to name individuals in the Acknowledgments.

Payment of Article Processing Charges (APC) does not qualify for authorship.

☒ Check here to confirm that all authors (including those to be added or removed) consent to the requested changes.

☐ ☒ Check here to confirm that you have read PLOS policies on Competing Interests and Funding Disclosures. Complete the followingtable to describe any updates needed to your article's disclosures.

	Updates needed to the submission's disclosure statement	No change needed
**Competing Interests**		☒
**Funding**		☒

Section 2. Final manuscript information

**Manuscript number**<br>e.g., PONE-D-17-00000	PONE-D-23-27550R3
**Complete author list, in correct order**<br>Please note any equal contributors with asterisks (*) or hashes (#)	Nayara da Silva Pontes, Sanderson José Costa de Assis, Gabrielle Silva de Oliveira, Rebeca de Castro Santana1, Rebeca Freitas de Oliveira Nunes, Emannuel Alcides Bezerra Rocha, Clécio G de Souza, Angelo Giuseppe Roncalli, Marcello Barbosa Otoni Gonçalves Guedes.
**Financial Disclosure** – including any additions/deletions necessary due to the change in authorship	This study did not receive any type of funding
**Competing Interests** – including any additions/deletions necessary due to the change in authorship	The authors declare no conflict of interest
**Acknowledgments statement**<br>Please acknowledge any removed authors if they contributed to the study in any way, as well as members of any author groups who do not meet our authorship criteria.	No thanks to be included

Section 3. Requests to add authors

Individual author addition #1

**Full name**	Emannuel Alcides Bezerra Rocha
**Email address**	emannuelrocha@yahoo.com.br
**Full affiliation**	Faculty of Health Sciences of Trairi, Santa Cruz, Federal University of Rio Grande do Norte, Rio Grande do Norte, Brazil.
**Briefly describe the reason for adding this author, and explain why they were not included on the submission previously.**	We requested the inclusion of Emannuel Alcides Bezerra Rocha as co-author of this article due to her indispensable contributions throughout the research process. Rocha was fundamental in formulating the hypotheses and methodology of the study, bringing an innovative approach that significantly enriched our work. His critical review of the manuscript helped to improve the clarity and coherence of the text, resulting in a substantial improvement in the presentation of the results.<br><br>Initially, Rocha was not included as a co-author due to a misunderstanding about her level of contribution and our attempt to follow the journal's authorship guidelines as accurately as possible. However, upon reviewing in more detail the contributions of each team member during the final stages of manuscript preparation, we recognize that Rocha fully met the authorship criteria established by the ICMJE, given her direct and substantial involvement in several critical steps of the study.<br><br>We believe that her inclusion as a co-author fairly and accurately reflects the contributions made and promotes transparency and scientific integrity."

Confirm that this person contributed to **all** of the authorship criteria for the relevant PLOS journal (see above).	X
**Tick all relevant boxes to indicate this individual's specific contributions:**	
Conceptualization	X
Data Curation	
Formal Analysis	
Funding Acquisition	
Investigation	
Methodology	X
Project Administration	
Resources	
Software	
Supervision	
Validation	
Visualization	
Writing – Original Draft Preparation	X
Writing – Review & Editing	X

Individual author addition #2 (if applicable)

**Full name**	Angelo Giuseppe Roncalli
**Email address**	roncalli@terra.com.br
**Full affiliation**	Public Health Program, Federal University of Rio Grande do Norte, Rio Grande do Norte, Brazil

**Briefly describe the reason for adding this author, and explain why they were not included on the submission previously.**	We requested the inclusion of Angelo Giuseppe Roncalli as a co-author of this article due to his substantial contributions in several phases of the project. Roncalli played a crucial role in developing the methodology and implementing the analytical techniques used in the study. He was also responsible for interpreting the results in detail and for contributing significantly to the writing and review of the manuscript.<br><br>Roncalli initial absence from the list of authors was due to an administrative error and a lack of clear communication about authorship criteria during the early phases of the project. As we move into the final stages of submission, we carefully review the contributions of everyone involved and conclude that Roncalli fully meets the authorship criteria established by the journal and the scientific community.<br><br>We recognize that its inclusion is essential to ensure that credit is distributed fairly and accurately, truly reflecting the contributions made. We believe that the addition of Roncalli as a co-author promotes scientific integrity and transparency."

Confirm that this person contributed to **all** of the authorship criteria for the relevant PLOS journal (see above).	X
**Specific contributions:**	
Conceptualization	X
Data Curation	X
Formal Analysis	X
Funding Acquisition	
Investigation	
Methodology	X
Project Administration	
Resources	
Software	
Supervision	
Validation	
Visualization	
Writing – Original Draft Preparation	X
Writing – Review & Editing	

Author group addition (if applicable)

**Group or consortium name**	
**Author who represents group**	
**Briefly describe the reason for adding this author group, and explain why they were not included on the submission previously.**	

Section 4. Requests to remove authors

Author removal #1

**Full name**	
**Email address**	

**Briefly describe the reason for removing this author at this time given their stated contributions to the manuscript and/or research.**	

**Which authorship criteria have NOT been met by this individual?** (Refer to the criteria numbers in Section 1 of this form)	
Are you updating the article's Acknowledgments to credit this person's contributions?	Yes
If yes, did they agree to be listed in the Acknowledgments?	Yes

If no, explain why you no longer wish to credit this individual for the work attributed to them in the Contributions listings provided previously.

Author removal #2 (if applicable)

**Full name**	
**Email address**	
**Briefly describe the reason for removing this author at this time given their stated contributions to the manuscript and/or research.**	

**Which authorship criteria have NOT been met by this individual?** (Refer to the criteria numbers in Section 1 of this form).	
Are you updating the article's Acknowledgments to credit this person's contributions?	Yes
If yes, did they agree to be listed in the Acknowledgments?	Yes
If no, explain why you no longer wish to credit this individual for the work attributed to them in the Contributions listings provided previously.	

Section 5. Requests for changes to author order

**Prior author list**	Sanderson José Costa de Assis, Nayara da Silva Pontes , Gabrielle Silva de Oliveira, Rebeca de Castro Santana, Rebeca Freitas de Oliveira Nunes, Emannuel Alcides Bezerra Rocha, Clécio G de Souza, Angelo Giuseppe Roncalli, Marcello Barbosa Otoni Gonçalves Guedes
**Requested author list**	Nayara da Silva Pontes, Sanderson José Costa de Assis, Gabrielle Silva de Oliveira, Rebeca de Castro Santana1, Rebeca Freitas de Oliveira Nunes, Emannuel Alcides Bezerra Rocha, Clécio G de Souza, Angelo Giuseppe Roncalli, Marcello Barbosa Otoni Gonçalves Guedes
**Briefly describe the reason for requesting a change in author order at this time.**	Initially, the order of authors was determined based on a preliminary understanding of individual contributions. However, after a more detailed review and discussion among all authors, we concluded that the new proposed order more fairly and accurately reflects the impact of each on the development of the study.<br><br>All authors agree with the proposed change and we believe it promotes transparency and scientific integrity by ensuring that credit is distributed in accordance with contributions made

**If any authors' contributions need to be updated, provide the affected authors' names and their updated contributions listings here.** Contributions should be listed using the CRediT Taxonomy terms listed below; list all terms that apply.<br><br>If no changes are needed, enter "N/A" in the response box.	

CRediT Taxonomy for author contributions

- Conceptualization
- Data Curation
- Formal Analysis
- Funding Acquisition
- Investigation
- Methodology
- Project Administration
- Resources
- Software
- Supervision
- Validation
- Visualization
- Writing – Original Draft Preparation

Documento assinado digitalmente
gov.br NAYARA DA SILVA PONTES
Data: 02/07/2024 18:43:01-0300
Verifique em https://validar.iti.gov.br

Nayara da Silva Pontes

Sanderson José Costa de Assis

Gabrielle Silva de Oliveira

Documento assinado digitalmente
gov.br REBECA DE CASTRO SANTANA
Data: 03/07/2024 20:02:10-0300
Verifique em https://validar.iti.gov.br

Rebeca de Castro Santana

Documento assinado digitalmente
gov.br REBECA FREITAS DE OLIVEIRA NUNES
Data: 04/07/2024 07:29:55-0300
Verifique em https://validar.iti.gov.br

Rebeca Freitas de Oliveira Nunes

Documento assinado digitalmente
gov.br EMANNUEL ALCIDES BEZERRA ROCHA
Data: 02/07/2024 18:26:06-0300
Verifique em https://validar.iti.gov.br

Emannuel Alcides Bezerra Rocha

Documento assinado digitalmente
gov.br CLECIO GABRIEL DE SOUZA
Data: 02/07/2024 21:30:33-0300
Verifique em https://validar.iti.gov.br

Clécio G de Souza

Angelo Giuseppe Roncalli

Documento assinado digitalmente
gov.br MARCELLO BARBOSA OTONI GONCALVES GUED
Data: 02/07/2024 16:44:32-0300
Verifique em https://validar.iti.gov.br

Marcello Barbosa Otoni Gonçalves Guedes
